# What Matters in a Relationship—Age, Sexual Satisfaction, Relationship Length, and Interpersonal Closeness as Predictors of Relationship Satisfaction in Young Adults

**DOI:** 10.3390/ijerph20054103

**Published:** 2023-02-24

**Authors:** Natalia Maja Józefacka, Elżbieta Szpakiewicz, Dominik Lech, Konrad Guzowski, Gabriela Kania

**Affiliations:** 1 Institute of Psychology, Pedagogical University of Krakow, Podchorążych 2, 30-084 Krakow, Poland; 2 Students Scientific Club ControlUP, Pedagogical University of Krakow, Podchorążych 2, 30-084 Krakow, Poland

**Keywords:** relationship satisfaction, sexual satisfaction, interpersonal closeness, young adults

## Abstract

Relationship satisfaction is one of the key elements affecting overall life satisfaction. This study aimed to identify significant predictors of relationship satisfaction in young adults in a romantic relationship. The study was questionnaire-based, involving 237 young adults who were currently in a relationship. Three self-rating scales were used: CSI-32 Relationship Satisfaction Scale, Sexual Satisfaction Questionnaire, and Unidimensional Relationship Closeness Scale. Sexual satisfaction proved to be a main predictor of relationship satisfaction in both sexes. For women, interpersonal closeness was additionally important, with a sense of closeness found to be even more important than sexual satisfaction for women cohabiting with their partners. Cohabiting people are generally more satisfied with their relationship, and a higher level of closeness and applied caresses can additionally be observed in them. In contrast, the relationship length appeared to matter only for men living with their partner: they were more satisfied with the relationship at the beginning of the relationship, and then their level of satisfaction declined. Relationship satisfaction in young adults appears to be determined by other factors depending on gender and cohabitation status. Nevertheless, at this age, sexual satisfaction proves to be one of the most critical factors for a sense of relationship satisfaction.

## 1. Introduction

The subject of close interpersonal relationships is widely discussed in the literature. The interest of researchers in this topic is not surprising, as high relationship quality is essential for the well-being of partners [1] and is also a determinant of happiness [2]. Satisfying social relationships may improve one’s mental and physical health [3]. Healthy romantic relationships are important for functioning in everyday life [4]. Partners in unsatisfying relationships tend to express emotions such as anger, criticism, and disgust more than satisfied couples [5], which negatively affects the well-being of both partners [6].

One of the key concepts relating to relationship satisfaction is Sternberg’s three-factor Theory of Love [7]. According to the theory, love consists of three elements: intimacy, commitment, and passion. Understood as a feeling of closeness and connection with the partner, intimacy gives a romantic relationship an experience of warmth [8]. Without this essential component, a feeling of emptiness and lack of desire to continue the relationship which is no longer satisfying begins to appear in the relationship after some time [9,10]. Commitment, also known as engagement, is constituted by conscious decisions to stay in a relationship and maintain it in the future. Commitment is shaped by actions taken, consisting of all kinds of efforts made to sustain the relationship and its stability, such as the decision to move in together. On the other hand, passion refers to physical attraction to one’s partner. The sexual aspect of a relationship is an element that is particularly noticeable in the early stages of a relationship. When sexual arousal begins to gradually diminish with increasing levels of stability, the relationship begins to lose its heat and becomes more of a friendship, which for many people is associated with a sense of burnout in the relationship [9,11]. In summary, Sternberg argues that all three components are necessary to experience full relationship satisfaction.

The goal of our study was to identify predictors of a satisfying relationship among young adults. Primarily, we have taken inspiration from Sternberg’s tri-factor Theory of Love, making an assumption that a satisfactory relationship is when all three Sternberg’s love components are present in a dyad. Other researchers take a slightly different approach in assessing relationship satisfaction. For example, Guerrero [12] finds that an important factor for relationship satisfaction is the perception of the attitude, behaviour, and communication of the partner with whom the individual is in a romantic relationship. However, others take an approach similar to ours and search for relationship predictors based on Sternberg’s theory. Closeness [13], as well as sexual agreement and relationships with others [14], also appear to be significant predictors of a good relationship. Some researchers search for differences in relationship satisfaction based on participants’ variables, such as gender or relationship length—they point to similar levels in perceived satisfaction between men and women [11,15] and relationships of varying relationship lengths [16]. Others mention cohabitation—Tai, Baxter, and Hewitt [17] indicate lower relationship satisfaction for couples not living together.

Eventually we decided to include passion, intimacy, and commitment, taken from Sternberg’s theory, as predictors of satisfying relationship. Furthermore, based on the literature review, we decided to broaden the initial list of satisfactory relationship components. As a result, we included participants’ age and relationship length as potential predictors of relationship satisfaction.

Commitment appears to be an essential factor for forming close relationships [18]. Researchers indicate that the decision to live with each other—although not the only factor—is an important component of commitment in a romantic relationship, which makes it more sustainable and more likely to grow [19,20]. Commitment theory provides insight into the motives behind the decision to move in together [21]. Partners voluntarily deciding to cohabitate begin to commit to the relationship and its continuation by sacrificing thinking about themselves and their needs in favour of partner-centred behaviours and motivations [22]. The results of a broad longitudinal study of American views on family issues indicated that almost two-thirds of American young adults consider cohabitation as a step in the courtship process (as of year 1998, which is almost 17 p.p. more than in 1986) [23]. Citing the author: “This endorsement of cohabitation as a prelude to marriage by more than three fifths of high school seniors is especially important because high school seniors are the primary individuals who will be most actively involved in making decisions about cohabitation and marriage in the coming years”. Furthermore, Monteiro et al. [24] found that commitment in dating and cohabitation relationships was higher than in dating relationships, where partners live apart from each other, though the results were not statistically significant.

We view the fact that partners are cohabiting as an indication of a high level of commitment among the partners. In his original article [7], Sternberg proposes another name for this component—decision. Thus, we conclude that viewing the decision to cohabit as a high level of commitment is justified.

Passion seems to be another essential factor. Sternberg describes passion as a drive perceived by a person, resulting in physical attraction to the partner, sexual intercourse, and romance [7]. Yela, in his Tetrangular Model [25]—which is an extension to Sternberg’s Triangular Theory of Love—differentiates passion into two components based on empirical findings. These passion components are erotic passion and romantic passion. Erotic passion refers to desires and needs of purely physiological nature, e.g., a rise in sexual arousal while being caressed by the partner. Romantic passion can be understood as psychological desires and needs, such as idealisation of the partner or the sense of romance felt in relationship [25]. Plopa [26], who created a reliable and valid psychometric tool for measuring sexual satisfaction (SSQ), understands passion similarly. In the questionnaire, the overall construct of sexual satisfaction is separated into three subscales: “sex” and “petting” (relating directly to satisfaction with physical contact), and “intimacy” (used to describe the more emotional and romantic sphere of sexuality). Research suggests that sexual satisfaction is important for relationship satisfaction in many cultures worldwide [16,27,28]. Given that relationship length in early adulthood is typically not very long, based on Sternberg’s notion that passion is most important in the early stages of a relationship, it can be assumed that sexual satisfaction will prove to be particularly important for young adults.

Despite sharing the same designation, intimacy regarded as a factor of sexual satisfaction is strongly related to passion, as it describes the feeling of closeness in romantic and sexual context. On the other hand, intimacy as an independent concept is conceptualized more broadly and refers to the feeling of closeness and bond in regard to the relationship as a whole, not only the sexual aspect (as described below).

Intimacy is usually understood as positive feelings and accompanying actions that produce attachment, closeness, and partners’ mutual dependence on each other [29]. Sternberg’s research indicates that intimacy understood this way consists of such components as, among others, the desire to care about the welfare of the partner, experiencing happiness in the presence of and because of the partner, respect for the partner, the belief that one can count on the partner in times of need, mutual understanding, mutual sharing of experiences and goods, giving and receiving emotional support, exchange of intimate information or mutual understanding and a sense of community—both material and spiritual. Surveys of Poles’ opinions on love [30] are identical to the conclusions of Sternberg’s concept. Thirty percent of respondents identify ‘true love’ with trust, loyalty to the partner and the resulting sense of security, striving for the other person’s well-being, or respect for the partner. It thus appears that love is often identified mainly with intimacy. We decided to use the URCS Questionnaire [31] as an operationalisation of Sternberg’s intimacy. Authors of the URCS questionnaire state the following: “In close, committed romantic relationships, for example, closeness and intimacy are likely to covary nearly perfectly and are conceptual twins” [31]. Therefore, we decided to conceptualize Sternberg’s intimacy as interpersonal closeness and assess its role in overall relationship satisfaction.

We decided to conduct research on young adults, as this stage of life is the time to face the Big Five life events [32]: leaving home, school completion, employment, marriage, and parenthood. Due to social changes that influence the amount of time necessary to complete the Big Five life events, the boundaries of young adulthood are now less rigid (and it is not easy to say when exactly young adulthood ends [33]).

The number of relationships is highest in young adulthood [34]. It is also the time when the level of emotional closeness in these relationships is higher than in any developmental period [35]. In young adults’ romantic relationships, which are influenced by early relationships with parents and peers [36], an important goal for individuals’ development is to create a healthy sense of sexuality [37]. In addition, compared to the earlier stages of childhood and adolescence, young adults can freely express their sexuality without anxiety or shame [38]. It is also a time when partners decide to move together as a sign of stability and commitment [39,40].

In this study, we wanted to understand what aspects are important for overall relationship satisfaction in young adults in a romantic relationship. There are many factors (e.g., good communication [41], attachment styles [42], personality [43], sociodemographic differences [44]) that predict relationship satisfaction, but we will focus on commitment (in form of cohabitation), passion, and intimacy from Sternberg’s Theory of Love and relationship duration. Based on previous research [45,46,47], we hypothesised that factors such as closeness, sexual satisfaction, and relationship length would be positively related to perceived relationship satisfaction. Additionally, cohabitation was singled out as an essential aspect—an indicator of the partners’ greater commitment to the relationship.

## 2. Materials and Methods

### 2.1. Participants

The research was conducted on a group of 237 young adults (76.79% women). The mean age of the respondents was M = 20.05 with SD = 1.65 (range = 18–25 years). The study was open to all students who remain in a heterosexual relationship longer than one month. The average relationship length was almost two years (M = 26.49 SD = 19.59), and there was no significant difference in relationship length between men and women (Z = −0.814, *p* = 0.416).

### 2.2. Data Collection

The data were collected via online social networking among college students and their friends. A convenience sampling approach was used among the Pedagogical University of Krakow and Jagiellonian University students who were encouraged to tell their friends to sign up to participate in the research. Participants completed the measures anonymously, providing background information about age, gender, relationship length, place of residence, and marital status.

### 2.3. Measurement

Participants were asked to answer three self-rating scales, using their PC or tablet. They were also asked to provide information on the age, gender, cohabitation status, and relationship length. We decided to divide participants based on gender and cohabitation status. Both URCS and SSQ validation results indicated gender differences in intimacy and sexual satisfaction, respectfully [26,31]. Moreover, Bühler in her meta-analysis [48] points out that results on gender differences in relationship satisfaction are inconclusive. Therefore, it is necessary to include gender as moderator to further investigate the matter; same applies to cohabitation status. Bühler suggests that it is important to examine the development of relationship satisfaction in couples that share the same household and in those who do not [48]. Diener [49] suggests that married couples who live apart are less satisfied than those who cohabitate, but there is not enough research conducted on informal relationships.

The Couples Satisfaction Index (CSI-32) developed by Funk and Rogge [50] (Polish adaptation by Stawska [51]) is a self-reporting questionnaire consisting of 32 items that measure relationship satisfaction. For each item (apart from the first one), participants are asked to respond on a six-point Likert scale (from 0 to 5 or reversed). Based on our research objective, we used the first two parts of this questionnaire. The reliability of this modified version of the scale was 0.82. The first part of the item evaluation stage consisted of 12 general statements that participants were asked to address, e.g., “I have a warm and comfortable relationship with my partner”, “I can’t imagine ending my relationship with my partner”. For each statement there were six possible responses, from “0—Completely not true” to “5—Completely true” (or reversed, for the two of the m). This item evaluation part also consisted of three items designed to measure the level of relationship disagreement, e.g., “in making major decisions” (responses from “5—We always agree” to “0—We always disagree”). In the last statement from this category, the participants were to consider the following: “Please indicate the degree of happiness, all things considered, of your relationship”. In this case, exceptionally, there were seven possible answers that ranged from “0—Extremely unhappy” to “6—Perfect”. The second part of this questionnaire consisted of eight more specific questions about the relationship that the participants were asked to answer on a six-point Likert scale (where “0” means “never”, “not at all”, etc., or reversed for the only one statement). In this part, statements were like the following: “In general, how often do you think that things between you and your partner are going well?”, “How rewarding is your relationship with your partner?”, “How well does your partner meet your needs?”, “Do you enjoy your partner’s company?”, “How often do you and your partner have fun together”? There was also one question regarding the social comparison: “How good is your relationship compared to most other relationships?”, with responses ranging from “0—Worse than all others (Extremely bad) to “5—Better than all others (Extremely good)”. 

The Sexual Satisfaction Questionnaire (SSQ) [26] consists of 10 items, with a five-point Likert response scale. Related sexual satisfaction is defined on the basis of three dimensions: intimacy, petting, and sex. The reliability level obtained for the overall scale score was 0.85. Example items from “intimacy” subscale were as follows: “Intimate conversations with partner”, “Perceiving the smell of your partner”, and “Hugging your partner”. Items from the “petting” subscale were as follows: “Caressing your intimate body parts by your partner” and “Caressing the intimate parts of the body of your partner with your hand”. Items from the “sex” subscale were, “Sexual intercourse with your partner” and “Experience orgasm during sexual intercourse with your partner”. Participants were to answer how satisfying each activity is for them. Answers ranged from: “0—Nonexistent”, through “1—No satisfaction” to “5—Maximal satisfaction”. Total score was a sum of all items.

Jobczyk’s Unidimensional Relationship Closeness Scale (URCS) [52] was used to measure interpersonal closeness conceptualized in this research as intimacy. It is a single-factor scale containing 11 statements such as, “I always consider X when making important decisions”, “I miss X when we are apart”, and “X and I want to spend time together”. It describes the overall sense of closeness on a seven-point Likert scale (1 = definitely no, 2 = no, 3 = rather no, 4 = hard to say, 5 = rather yes, 6 = yes, 7 = definitely yes). The result is the sum of scores from all 11 items; a higher note means a higher sense of closeness in the relationship with X. Although the scale is designed to assess closeness in interpersonal relationships in general, we decided to use it to research romantic relationships. Our decision was based on the capacity of this scale to match previous models assessing romantic closeness of couples (e.g., Dibble et al. [31]). The reliability level was 0.97.

### 2.4. Statistical Analysis

To define the connection between gender, cohabitant status, and psychological factors of relationship satisfaction, statistical analysis with the use of the 26 SPSS programme was performed.

In the first order, the reliability of all scales used in the study was assessed. Descriptive statistics (Table 1), as well as Shapiro–Wilk’s normality test, were calculated. The central limit theorem was used to deal with the sampling distribution’s normality assumption, where possible.

The next measure investigated the existence of gender differences and differences in the psychological factors analyzed between couples living together and separately, as well as also checking for correlation between variables in both groups (gender, place of living). For this purpose, the U Mann–Whitney test and r Pearson correlation were used. This non-parametric test was chosen because the assumption of equinumerosity of groups was violated in both analyses.

The final measure examined which psychological factors best explained relationship satisfaction in the subgroups distinguished based on the gender and residence of both partners.

## 3. Results

The results of Cronbach’s α, ω analysis indicated a sufficient level of reliability in all questionnaires. The individual measures are reported together with a description of the scales. Descriptive statistics were calculated for age, relationship length, and other psychological factors; detailed results are provided in Table 1.

The analysis of intergender differences performed with the U Mann–Whitney test demonstrated that in most variables, there were no differences between genders (Table 2). The only significant difference was in the sense of closeness (URCS), which was higher in women. It is worth noting that the two subgroups did not differ significantly in terms of relationship length and age, indicating that the groups were equivalent in this respect. The analysis of correlation matrix (Table 3) indicates significant correlation between relationship satisfaction and sexual satisfaction in both genders. Additionally, only in the women subgroups was Closeness significantly related with relationship satisfaction and sexual satisfaction.

The next measure examined differences between people living together and separately (Table 4) as well as correlation between variables in both scenarios (Table 5). Significant differences concerned feelings of closeness (URCS) and overall relationship satisfaction (CSI). In all cases, higher scores were found in the group of people living with a partner. The effect strength was low. As expected, there were differences both in the respondents’ age and relationship length. Those declaring to live together with a partner are statistically older and have longer relationship length. In case of people declaring living separately, all analysed psychological variables were significantly correlated. For couples living together, only relationship satisfaction and sexual satisfaction were correlated.

To further deepen the analyses, it was decided to see which factors are the primary relationship satisfaction predictors when broken down by gender and people living together or separately. Stepwise regression analysis was used. Relationship length, subject age, interpersonal closeness, and sexual satisfaction were selected as predictors (Table 6).

The performed regression analysis demonstrated that in all conditions analysed, sexual satisfaction is an essential factor in relationship satisfaction. The sense of closeness is an equally important aspect in women, present both in couples living together and couples living separately. It is advisable, however, to note the value of the standardised regression coefficient (beta) for the sense of closeness in women in both conditions analysed. For the first condition—those not living together—closeness is an important, but not the key, predictor. By contrast, in the second condition, its strength is much stronger. The above model also better predicts relationship satisfaction in the male group than in the female group.

## 4. Discussion

The conducted study captures important components associated with satisfaction in a romantic relationship in young adults in the context of the three-factor theory of love [7]. According to Levinson [53], it is during early adulthood that we enter the adult world, connecting with society and realising ourselves through the establishment of mature interpersonal relationships. Based on the results of the conducted research, it can be seen that women value the issue of closeness more.

Cohabitation has become a normative stage of entering adulthood. Relatively little is known about how young adults decide to enter concubinage, when in their relationships this transition occurs, and what such a step means for them [48,54]. Many young people express a belief that living together will help them choose a better life partner for marriage [23,55]. Surra’s research [56,57,58] indicates that there are two main reasons for the decision to move in together: (a) related to circumstances, e.g., need for housing, change of employment, life costs; (b) related to the development of commitment. Based on the results of the present project, it can be seen that young adults who have decided to cohabit also indicate in line with the second reason Surra shows: higher levels of relationship satisfaction. That is why our results shows that the decision to cohabit may be an indicator of greater commitment to the relationship. Nevertheless, there is little research shedding light on the predictors of the decision to cohabit. It is certainly an important theme worth pursuing in future studies.

The final outcome of the analyses was the examination of the relationship satisfaction predictors. It was assumed that these could be intimacy and sexual satisfaction. In all analysed subgroups, sexual satisfaction was the most crucial factor. Sexual satisfaction, presented in our study as a component of passion according to Sternberg’s theory, is indeed vital for a successful relationship. The findings described are furthermore consistent with the tetrangular theory of love [25] proposed as an extension of Sternberg’s three-factor theory. Indeed, the SSQ used in the study differentiates from the tetrangular theory in the construct of sexual satisfaction into physical (petting, sex) and emotional (intimacy) issues [26]. The ability to form and maintain sexual relationships with others is sometimes seen as the most critical skill for individual development in early adulthood [37]. During early adulthood, in contrast to earlier stages of development, the individual can freely express their sexuality without significant fear or guilt [59].

It is worth noting that for women, interpersonal closeness appears among the predictors of relationship satisfaction. We can see an intergender difference here, as for men, sexual satisfaction is the only significant predictor.

Furthermore, for women not living with a partner, sexual satisfaction is the main predictor of relationship satisfaction with beta = 0.49, with interpersonal closeness coming second with beta = 0.17. In contrast, for women living with a partner, interpersonal closeness becomes the strongest predictor with beta = 0.52, where sexual satisfaction comes second with beta = 0.33.

We can see that the relationship satisfaction’s structure is different in women who cohabit with their partner, in comparison to those who do not.

The Basson’s Female Sexual Response Model [60,61] seems to be helpful in explaining this phenomenon. Bassonn’s model assumes that with the passage of time and the development of the relationship, women’s motivation to experience sexual intercourse is mainly determined by the desire to increase intimacy between partners [62]. However, the pleasurable physical experience of “being sexual” is important for this intimacy motivation to persist in the long term.

On the contrary, at the early stages of relationship, when passion between partners is the dominant factor characterising their relationship [7], sexual response among women is mainly triggered by physiological arousal—as in the linear model of sexual response [60]. This linear model of sexual response is also relatively effective for describing men’s sexuality [60]. Our results are consistent with Basson’s line of argumentation. Sexual satisfaction is an important predictor of relationship satisfaction in both genders, both cohabiting and non-cohabiting. However, in women who are in more committed relationships (i.e., when individuals live together), relationship satisfaction is strongly related to interpersonal closeness, with sexual satisfaction still present among its predictors.

## 5. Conclusions

Women place a higher value on experienced closeness. Higher levels of closeness, applied caresses, and overall relationship satisfaction can be observed in the cohabiting couple. Regardless of gender and cohabitation, for all subgroups analysed, sexual satisfaction was an important factor in relationship satisfaction.

The relationship between the importance women placed on intimacy and sexual satisfaction differed between women living with their partners or living apart. At the earlier stage of the relationship, sexual satisfaction is the main predictor of relationship satisfaction. These results are consistent across both sexes. In contrast, at later stages, where partners live together, a sense of closeness becomes a more important factor for women, although sexual satisfaction continues to be important.

### Limitation

The survey was made available on Facebook in the form of online questionnaires—the sample was collected using the virtual snowball sampling method. Research suggests that this method allows large and representative groups to be obtained [61]. However, it was impossible to supervise the conditions under which the questionnaire was completed. For this reason, it would have been advisable to carry it out in direct contact with the respondents and to use methods other than self-reporting, e.g., experimental. It is worth to notice that it is a cross-sectional/correlational study and causality cannot be inferred.

When repeating the survey in the future, it would be worth taking into account several aspects, such as selecting groups with a higher representation of men, examining couples with longer relationship length, checking the length of cohabitation, marital status, or/and the channels through which the couples met (e.g., through friends, common activities, dating apps, etc.). It would have been appropriate to investigate specific personality traits and their possible impact on the variables investigated (intimacy, commitment, and passion); this would have led us to more complete statistical analyses, which would have facilitated a clear distinction between predictor and/or mediator variables of couple satisfaction. Combining couples’ responses would be beneficial for comparing the average scores of the individual partners.

In the literature, many researchers also point to gender differences not taken into account in this study [62,63,64], as well as some issues of individual development [65]. The aspects mentioned above may prove relevant in the construction of further research projects.

## Figures and Tables

**Table 1:** Descriptive statistics and normality tests for psychological factors with Shapiro–Wilk test.

Variable	M	SD	Me	Min	Max	Skew.	Kurt.	W	*p*
Age	20.50	1.66	20	18	25	0.99	0.34	0.87	<0.001
Relationship Length (month)	26.49	19.59	18	1	96	1.04	0.54	0.90	<0.001
URCS	62.16	9.30	65	0	66	−4.71	25.45	0.43	<0.001
SSQ	39.96	7.15	41	0	50	−1.54	4.59	0.90	<0.001
CSI-32	85.61	11.50	89	31	102	−1.59	3.36	0.87	<0.001

Note: M—mean, SD—standard deviation, Me—median, Min—minimum, Max—maximum, Skew.—skewness, Kurt.—kurtosis, W—Shapiro–Wilk’s test statistic.

**Table 2:** Intergender differences in individual variables.

	Man (*n* = 53)				Woman (*n* = 184)					
Variable	Mean Range	*Me*	*Min*	*Max*	Mean Range	*Me*	*Min*	*Max*	*p*	η2
Age	134.74	21.0	18	25	114.47	20.00	18.00	25	0.051	0.02
Relationship Length (month)	93.09	1.5	0	18	100.80	1.50	0.00	45	0.416	<0.01
SSQ_TOTAL	115.45	40.0	0	50	120.02	41.00	12.00	50	0.669	<0.01
URCS	91.38	63.0	4	66	126.96	66.00	0.00	66	<0.001	0.05
CSI-32	113.43	89.0	31	102	120.60	89.00	38.00	102	0.502	<0.01

**Table 3:** Intergender correlation matrix.

Men		Relationship Length	URCS	CSI
URCS	*p*	0.11		
	*r*	0.466		
CSI	*p*	0.08	0.15	
	*r*	0.592	0.282	
SSQ	*p*	0.02	0.13	0.76
	*r*	0.881	0.355	<0.001
Women				
URCS	*p*	0.01		
	*r*	0.910		
CSI	*p*	−0.05	0.35	
	*r*	0.564	<0.001	
SSQ	*p*	<0.01	0.29	0.52
	*r*	0.974	<0.001	<0.001

**Table 4:** Differences in psychological factors in people living together or separately.

	Live Separately (*n* = 166)				Live Together (*n* = 71)					
Variable	Mean Range	Me	Min	Max	Mean Range	Me	Min	Max	*p*	η2
Age	110.55	20.0	18	25	138.76	21.00	19.00	25.00	<0.01	0.04
Relationship Length	91.28	1.5	0	25	117.05	2.00	0.50	45.00	<0.01	0.04
SSQ_TOTAL	114.78	40.0	0	50	128.87	42	23	50	0.147	0.01
URCS	112.56	65.0	0	66	134.06	66	4	66	<0.05	0.02
CSI-32	112.13	88.0	31	99	135.07	91	65	102	<0.05	0.02

**Table 5:** Correlation matrix in psychological factors in people living together or separately.

Living Separately		Relationship Length	URCS	CSI
URCS	*p*	0.05		
	*r*	0.557		
CSI	*p*	0.12	0.33	
	*r*	0.172	<0.001	
SSQ	*p*	0.05	0.31	0.60
	*r*	0.550	<0.001	<0.001
Living together				
URCS	*p*	−0.03		
	*r*	0.809		
CSI	*p*	−0.39	0.12	
	*r*	0.002	0.308	
SSQ	*p*	−0.11	0.01	0.51
	*r*	0.394	0.897	<0.001

**Table 6:** Relationship satisfaction predictors.

Living	Gender	*n*	Predictors	*B*	*SE*	*Beta*	*t*	*p*	*R* ^2^	*F*	*p*
Separately	Men	34	(Constant)	37.91	7.15		5.30	<0.001	0.54	39.80	<0.001
			SSQ	1.16	0.18	0.74	6.31	<0.001			
	Women	132	(Constant)	40.53	6.08		6.66	0.000	0.31	30.37	<0.001
			SSQ	0.82	0.13	0.49	6.34	<0.001			
			URCS	0.19	0.08	0.17	2.28	0.024			
Together	Men	19	(Constant)	38.16	10.57		3.61	0.002	0.55	23.14	<0.001
			SSQ	1.19	0.25	0.76	4.81	<0.001			
	Women	71	(Constant)	−49.44	24.47		−2.02	0.049	0.45	19.86	<0.001
			SSQ	0.44	0.14	0.33	3.09	0.003			
			URCS	1.85	0.38	0.52	4.84	<0.001			

## Data Availability

Not applicable.
